# Connected diagnostics: linking digital rapid diagnostic tests and mobile health wallets to diagnose and treat brucellosis in Samburu, Kenya

**DOI:** 10.1186/s12911-019-0854-4

**Published:** 2019-07-22

**Authors:** S. Smith, R. Koech, D. Nzorubara, M. Otieno, L. Wong, G. Bhat, E. van den Bogaart, M. Thuranira, D. Onchonga, T. F. Rinke de Wit

**Affiliations:** 1 PharmAccess Foundation, AHTC Tower 4C, Paasheuvelweg 25, 1105 BP Amsterdam, The Netherlands; 2 PharmAccess Foundation Kenya, New Rehema House, Rhamta Road, Westlands, Nairobi, Kenya; 3 Fio Corporation, 111 Queen Street East Suite 500, Toronto, Ontario M5C 1S2 Canada; 4 Mondial Diagnostics, Meibergdreef 39, 1105 AZ Amsterdam, The Netherlands; 5 Samburu County Government, C77, P.O. Box 3 – 20600, Maralal, Samburu County Kenya

**Keywords:** Diagnostics, Brucellosis, Malaria, mHealth, Mobile health, Mobile health wallet

## Abstract

**Background:**

Despite WHO guidelines for testing all suspected cases of malaria before initiating treatment, presumptive malaria treatment remains common practice among some clinicians and in certain low-resource settings the capacity for microscopic testing is limited. This can lead to misdiagnosis, resulting in increased morbidity due to lack of treatment for undetected conditions, increased healthcare costs, and potential for drug resistance. This is particularly an issue as multiple conditions share the similar etiologies to malaria, including brucellosis, a rare, under-detected zoonosis. Linking rapid diagnostic tests (RDTs) and digital test readers for the detection of febrile illnesses can mitigate this risk and improve case management of febrile illness.

**Methods:**

This technical advance study examines Connected Diagnostics, an approach that combines the use of point-of-care RDTs for malaria and brucellosis, digitally interpreted by a rapid diagnostic test reader (Deki Reader) and connected to mobile payment mechanisms to facilitate the diagnosis and treatment of febrile illness in nomadic populations in Samburu County, Kenya. Consenting febrile patients were tested with RDTs and patient diagnosis and risk information were uploaded to a cloud database via the Deki Reader. Patients with positive diagnoses were provided digital vouchers for transportation to the clinic and treatment via their health wallet on their mobile phones.

**Results:**

In total, 288 patients were tested during outreach visits, with 9% testing positive for brucellosis and 0.6% testing positive for malaria. All patients, regardless of diagnosis were provided with a mobile health wallet on their cellular phones to facilitate their transport to the clinic, and for patients testing positive for brucellosis or malaria, the wallet funded their treatment. The use of the Deki Reader in addition to quality diagnostics at point of care also facilitated geographic mapping of patient diagnoses in relation to key risk areas for brucellosis transmission.

**Conclusions:**

This study demonstrates that the Connected Dx approach can be effective even when addressing a remote, nomadic population and a rare disease, indicating that this approach to diagnosing, treatment, and payment for healthcare costs is feasible and can be scaled to address more prevalent diseases and conditions in more populous contexts.

## Background

Febrile illness is a leading cause of morbidity and mortality across sub-Saharan Africa and the most common reason for seeking medical care amongst African children [1–3]. Historically attributed to malaria, febrile illness is increasingly recognized as a disease of multiple etiologies, with viral- and bacterial-associated fevers prevailing over malaria in many African settings [4–7]. Unfortunately, despite this considerable reduction in the proportion of malaria-associated fevers [5, 8] and the World Health Organization (WHO) recommendation to test all suspected malaria cases before providing treatment [9], presumptive treatment of malaria remains a consolidated practice amongst clinicians working in malaria-endemic areas, exposing patients to the risk of misdiagnosis and its consequences thereof [10–13]. These include not only over-prescription of antimalarial drugs, with potentially negative effects on patient health and subsequent parasite drug resistance, but also a delay in achieving the correct diagnosis, which can prove fatal, as confirmed by the high mortality rates associated with non-malarial fevers [13–16]. In low-resource settings, management of febrile patients is complicated primarily due to the lack of accessible diagnostic tools to correctly identify causes of non-malarial fever [17, 18].

An example of one such febrile illness that is often misdiagnosed as malaria is brucellosis. Brucellosis is a zoonotic disease caused by ingestion of unpasteurized milk, blood or undercooked meat from infected animals, or close contact with their secretions. Brucellosis is often misdiagnosed and/or inappropriately treated due to lack of public awareness and adequate diagnostic capabilities in the rural facilities which do not have functional laboratory services [19]. A study conducted in two hospitals in Northern Tanzania found that, of febrile patients misdiagnosed with malaria, 3.1% had brucellosis [20]. The disease, while less common than malaria, is widespread across many African countries, including Kenya where the estimated seroprevalence is 3.0% [19]. The disease, when left untreated, can cause severe illness and economic disruption.

Whilst the roll-out of rapid diagnostic tests (RDTs) has simplified diagnosis of malaria at all levels, recognition of other tropical infections such as brucellosis remains challenging, as it often requires a central laboratory facility which many rural, resource-poor areas lack [17, 18] and/or relies on often poorly performed assays [21]. Furthermore, for those in rural areas, the costly need for repeat health facility visits (including visits related to testing, diagnosis, and treatment) that often occur over a period of weeks can result in lack of patient trust in the healthcare system, and increases the risk of patients remaining untreated for their illnesses [22]. These constraints highlight the need for simple, high-quality tests for point-of-care (POC) diagnosis of non-malarial febrile illnesses such as brucellosis as part of an affordable, integrated healthcare service.

To date, nearly 5 billion people around the world own a mobile phone, a fifth of whom reside in Africa [20]. Considered as one of the greatest social equalizers of our time, mobile phones do not only empower people by enabling them to communicate with each other and exchange information; they also are revolutionizing the way financial and health care services are delivered to underserved communities, promoting financial inclusion and health savings [23–25]. In Kenya, one of the world’s leaders in mobile money thanks to the M-Pesa system, a digital platform for financial inclusion in healthcare, called M-TIBA, was launched in 2015 [25]. M-TIBA is meant to serve the needs of Kenyans, and in particular those who do not have access to traditional banking and financial services, nor to adequate basic healthcare for themselves and their families. These people have to either rely on the already overstretched public health services or turn to the private sector and pay for healthcare out-of-pocket. M-TIBA offers a sustainable solution by enabling Kenyan citizens to save, send, receive and pay money for medical treatment through a dedicated mobile health wallet on their phone. Because use of these funds is restricted to conditional spending at selected quality-controlled healthcare providers, M-TIBA promotes healthcare inclusion among all levels of society, expanding access to health saving accounts and opening up innovative mechanisms of healthcare funding. Furthermore, donors and insurers can use M-TIBA as a transparent tool for offering healthcare financing products (e.g. vouchers and low-cost health insurance) to specific segments of the Kenyan population.

Banking on the Africa’s recent mobile phone revolution, and in light of the above challenges with respect to more efficiently managing febrile illnesses, we designed a novel intervention aimed at improving the cost-effectiveness of care provision. This ‘Connected Diagnostics’ (Connected Dx) approach enables febrile patients in remote parts of Africa to connect, not only clinically but also financially, to the local healthcare system. Our approach leverages on the ability to integrate rapid POC diagnostics, mobile telephone networks, and bank-less banking systems into a virtual circuit that centers on the patients and provides them with easier access to pertinent diagnostics and quality medical services. In essence, the program seeks to i) identify febrile patients, ii) test them for malaria and brucellosis using RDTs interpreted by a mobile network-connected RDT test reader and, iii) based on accurate point-of-care diagnosis, disperse funds in semi-real time to patients’ mobile health wallets to enable access to appropriate treatment and follow-up care. This intervention is expected to promote increased use of WHO-approved diagnostics (in this instance, RDTs) amongst febrile patients, reduce misdiagnosis, and contribute to cost savings that will promote greater transparency and efficiency across different parts of the healthcare system. In addition, the adoption of a cloud-based electronic data capture system provides up-to-date geographic insights into the local disease prevalence and incidence, enabling authorities and healthcare policy makers to design interventions accordingly and to promptly detect potential outbreaks.

In this study, the feasibility and acceptability of the proposed intervention was evaluated. To assess the Connected Dx concept under extreme conditions, the program was piloted in a remote region in rural Kenya (Samburu County) amongst an ambulant nomadic community with known brucellosis transmission [26]. The Samburu County Referral Hospital reported 2228 positive cases of brucellosis from 2014 to 2016 [27]. Brucellosis is both preventable and treatable with usually available antibiotics (like ciprofloxacin-doxycycline and cotrimoxazole), but can result in poor outcomes in humans including adverse pregnancy outcomes [28, 29], arthritis, hepatomegaly (enlargement of liver), endocarditis (enlargement of heart), and various neurological syndromes [30].

## Methods

### Study area

The study was conducted in Samburu County, Northern Kenya, through the local healthcare facilities of Maralal, Kisima and Suguta Marmar and their surrounding areas. This region is remote but well-connected due to two GSM masts located in the town of Maralal and its nearby airstrip, which provide internet connection to the area and well-functioning mobile phone network coverage. Samburu County, a semi-arid and sparsely populated area in the Rift Valley region, is home to 223,947 people, the majority of whom are semi-nomadic pastoralists belonging to the Samburu tribe [31]. Its capital and largest city, Maralal, was originally established as a livestock market and is now a major thriving center for the local population. Samburu County is the second poorest county in Kenya, with approximately 80% of the population living below the poverty line [31]. Like other pastoralist tribes in Kenya, the Samburu community rely on livestock for their livelihood (mainly cattle, goats, sheep and camels), which they use as main source of food and income. Consumption of raw meat and blood and fresh milk, improper disposal of animal materials, poor hygienic conditions during calving and slaughtering, frequent intermingling of herds at the few available water points, along with the absence of a control program contribute to the local spread of brucellosis. Human brucellosis prevalence rates of approximately 2% were reported in Samburu Central Sub-County [27] and livestock seroprevalence rates have been estimated at 10.7–14.9% [19]. It is generally understood that, in regions with high livestock seroprevalence of brucellosis, human brucellosis is likely underdiagnosed [32]. The estimated malaria prevalence within the county is 2.6% [27].

Access to healthcare services in Samburu is poor as a result of various factors, including difficulties in reaching healthcare facilities (due to long distances, poor roads, lack of money for transportation, regional insecurities, etc.), poverty, cultural practices, lack of information, poor health-seeking behaviours and poor-quality health services [33]. Currently, the town of Maralal hosts a referral hospital for about 95 health posts and clinics in the Central Samburu Sub-County, featuring a staff of over 100 people, including a laboratory and pharmacy. The process for diagnosis, treatment, and case management of brucellosis is lengthy and results in significant loss-to-follow-up for diagnosed patients. Diagnosis of brucellosis using serological testing can only be performed at a limited number of labs in the entire county [34] and can include a variety of testing (included but not limited to Enzyme Linked Immunosorbent Assay (ELISA) test, Rose Bengal plate tests (RBPT), Complement fixation test (CFT), and recent Polymerase Chain Reaction (PCR) [35]. Sensitivity and specificity of these testing methods vary and are largely dependent on proficiency of lab technicians performing the test. Due to test procedures, necessary incubation times, and significant demand on the limited Samburu healthcare system, the length of time from testing to diagnosis can be several days, requiring patients to make multiple trips to the clinic between testing and dispensing of treatment. This can lead to significant gaps in patient care management, which can lead to crucial impact on disease morbidity when considering the nomadic nature of the Samburu population.

### Study design and objectives

From September 2016 to April 2017, a feasibility study was conducted aimed at assessing acceptability, usability, and clinical outcomes of a mobile-phone based intervention linking on-site RDT for brucellosis and malaria through dedicated readers with digital financing and health care provision measures. This mHealth intervention relied on the use of a novel mobile-based money transfer system (the “mobile health wallet”) to finance access to appropriate medical care combined with short messaging services (SMS) to improve uptake and sustained use of healthcare services, promote patient’s adherence to treatment and alert medical professionals at a central medical facility for the presence of patients in their catchment area. To demonstrate the feasibility of this novel financing approach, this study targeted brucellosis, an important but often underdiagnosed cause of febrile illness in Samburu, for which a high-quality, well-validated POC test has been developed [36, 37].

### Study population and inclusion criteria

Participants to the study were initially recruited (September–December 2016) amongst patients with fever who self-reported at the outpatient clinics of Maralal, Kisima and Suguta Marmar or who were mobilized via community outreach interventions that took place in the study area. Subsequently (February–April 2017), recruitment efforts mostly focused on people at high risk of contracting brucellosis (e.g., butchers, slaughterers, household contacts of brucellosis patients, etc.), through targeted outreaches and household contact tracing. Part of the outreach activities were integrated with other ongoing initiatives (‘Beyond Zero Campaign’, ‘Trachoma Elimination Project’), in close collaboration with the Kenyan Ministry of Health and AMREF. Social mobilization was conducted within 3 days prior to actual outreach by the Health Promotion Department of the County Health Office. Key health messages were disseminated by Community Health Volunteers (CHVs) during the local administration meeting (Chief Barazas) and dialogue days held by Community Health Extension Workers.

Consenting patients were included in the study if they (or any of their household members) owned a mobile phone operated by Safaricom telecom operator (Safaricom has the largest market share of Kenyan mobile operators), and if they suffered from fever (defined as a forehead-scanned body temperature > 37.5 °C), measured either on admission as measured by a scanner (RoHS body infrared thermometer) during outreach visits or self-reported during the days before admission, and/or from any of the following self-reported symptoms: (night) sweats, fatigue, weight loss, nausea and/or vomiting, headache, general discomfort, joint pain and lower back pain. The lower minimum temperature for fever (> 37.5 °C as opposed to the standard > 38.0 °C for rectal/ear temperature) was used to accommodate for inaccuracies in non-contact infrared thermometers (±0.5°-1 °C).

### Study procedures

**Fig. 1:**
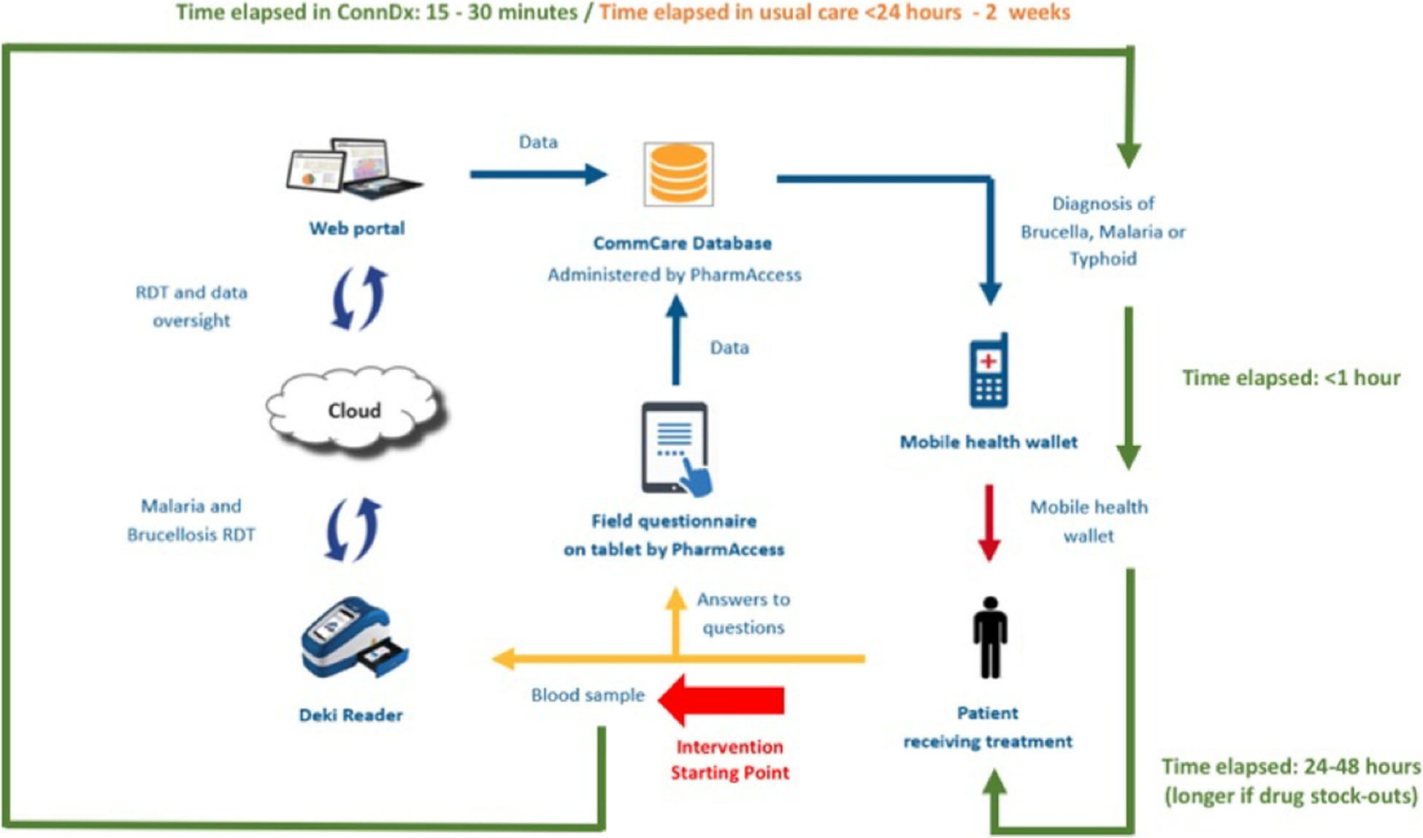
Algorithm summarizing the study procedures and timeline, and in particular comparing this timeline to that of usual care

The study procedure is visualized in Fig. 1. It involved the following steps:

#### Diagnostic testing and patient management

Consenting patients meeting the inclusion criteria were enrolled in the study by CHVs, who recorded patient’s symptoms and body temperature on an electronic Case Record Form (CRF). After enrolment, patients underwent serological testing for brucellosis using lateral flow assays (LFAs) performed on-site by a trained health worker (nurse or laboratory technologist). Diagnosis of malaria was based on detection of circulating *Plasmodium falciparum*-specific histidine-rich protein II (HRP-2) by *CareStart*™ Malaria HRP2 (Pf) RDTs (IFU G0131, Access Bio, Somerset, USA), as routinely done at Maralal Hospital. Diagnosis of brucellosis was based on detection of antibodies by the MondialDx Brucella Human IgM/IgG LFA (Mondial Diagnostics, Amsterdam, The Netherlands). Diagnosis for brucellosis was confirmed with positive reaction in either or both IgM and IgG (given the knowledge that, although reaction for IgG alone can indicate a persistent brucellosis infection, acute infection cannot be determined serologically [37]). RDTs were performed according to the manufacturer’s recommended procedures. Blood was collected from a brief finger-prick with the aid of disposable pipettes and tips and dispensed in appropriate volumes to the corresponding RDTs, immediately followed by 2 to 5 drops of the supplied assay buffer solution. After 15 to 20 min from the initial application of the specimen, the RDTs were interpreted (for positive diagnosis, negative diagnosis, or error) and recorded by the Deki Reader™ device (Fio Corporation, Toronto, Canada). The Deki Reader™ device is an automated reader clinically validated for use with various RDTs, including malaria RDTs across several malaria-endemic countries, including Kenya [38–40]. Its overall performance with respect to test result interpretation, ability to reduce end-user diagnostic errors through a step-by-step job aid, and quality of data collection and storage via a cloud-based web portal has been demonstrated in a number of field deployments across Africa [38–41]. For the purposes of this study, the Deki Reader™ was also calibrated to recognize and interpret the MondialDx Brucella Human IgM/IgG LFA. The device was used to: i) assist the users in performing the RDTs correctly with in-process quality control features to detect and correct user errors on-the-spot, ii) keep track of the incubation time for each RDT processed, iii) provide an automated interpretation of malaria test results, and iv) capture an image of every RDT processed at the time of interpretation and upload it onto the project’s mobile phone cloud-database (CommCare^1^) for review. Patients positively diagnosed with brucellosis were referred to the clinics of Maralal, Kisima or Suguta Marmar to conduct further follow-ups. This included confirmatory testing, on-site treatment and referrals to nearby clinics for treatment. For malaria, a co-formulation of artemether (20 mg) and lumefantrine (120 mg) to be administered as a 6-dose regimen over 3 days was prescribed, according to the national guidelines [42], while treatment for brucellosis in adults and children consisted of a short-course (5 days) of oral ciprofloxacin-doxycycline and cotrimoxazole, respectively [43–45]. The choice of administering a quinolone-containing regimen combined with doxycycline, instead of the most commonly used rifampicin, reflected Kenyan National Policy of restricting the use of rifampicin to patients affected by tuberculosis (HIV/TB co-infected patients) [46, 47].

#### Data collection and analysis

After enrolment, patients were questioned on-site regarding their social, economic and educational background, and their attitudes and practices with respect to disease knowledge and exposure risks. Patients’ age and gender, along with their identity code, GPS location at the test site, test results, and images of processed RDTs were collected through the Deki Reader™ devices, uploaded onto a web portal (Fionet™), and linked to the CommCare cloud-database for further analysis. In addition, detailed information on patient socio-demographic (age, household position, education level, occupation, income, housing, etc.) and behavioral features (use of bed-nets, food habits, hand washing and contact with livestock) were digitally collected by means of structured questionnaires administered by CHVs in the local language. The resulting two datasets were subsequently consolidated in a web-based database through a unique patient identifier that was automatically assigned to each study participant and used for all data collection procedures. A follow-up questionnaire was administered by phone to patients positively diagnosed with brucellosis to assess their self-reported satisfaction with the intervention and evaluating its impact on patient health and disease knowledge. Descriptive analyses (conducted using STATA statistical software) were conducted to describe the overall study population, assess the prevalence of brucellosis amongst the study participants, and identify the prevalence potential risk factors associated with brucellosis in the population. Univariate analyses (Chi-Square tests) were conducted to determine associations between commonly known risk factors for brucellosis transmission and patient diagnosis. Furthermore, geographic (GIS) analysis was conducted using ArcGIS to determine potential associations between location of brucellosis diagnosis (as recorded by the Deki Reader™) and proximity to brucellosis risk areas (butcheries, slaughterhouses, cattle markets, etc.).

#### Mobile health wallet

By uploading of a positive test result for malaria or brucellosis onto the CommCare cloud-database, the Deki Reader™ device automatically triggered a cascade of events that culminated in the deposit of a brucellosis treatment voucher into the patient’s mobile health wallet. As such, treatment vouchers were only provided to patients with a positive POC diagnosis. This mobile health wallet is comprised of an Unstructured Supplementary Service Data (USSD)-based interface (front-end) that beneficiaries use to transact with the financial system, send and receive information, and make payments, as well as a clearing-house infrastructure at the back-end which provides linkage to the mobile money transfer system M-Pesa. The clearing-house infrastructure collects real-time data on payments and on medical utilization and is responsible for handling all administrative duties, record-keeping, reconciliation of funds, etc. The design of the mobile health wallet ensured that patients positively diagnosed with malaria or brucellosis were able to use these vouchers only in exchange for medical treatment and follow-up care at the health centers of Malaral, Kisima or Suguta Marmar, and for transport to and from the facilities. To encourage health-seeking behaviors even in those testing negative for brucellosis, this mobile health wallet was subsequently extended to all febrile patients enrolled in the second phase of the study to facilitate transport to the health centers for further testing. A system of SMS text messages informed the patients about their condition and enabled the physicians to send treatment reminders and eventually follow up on treatment progress and outcome. A visual depiction and timeline of the Connected Dx-facilitated brucellosis case management process, in comparison to the usual approach within Samburu, is provided in Fig. 1.

### Ethical considerations

The study procedure (and all databases used within the study) was approved by the Samburu County Government and the Kenyatta National Hospital/University of Nairobi Ethics and Research Committee (Ethical Clearance Nr. KNH-ERC/RR/401). Written informed consent was obtained from each study participant (or parent/guardian in the case of minors under 18 years of age), after providing information on the study aim and procedures in the local language. Due to the common practice of phone-sharing within Samburu families and households, patients (or parent/guardian in the case of minors under 18 years of age) were informed during the consent process that any diagnoses would be sent to their mobile phone via an SMS message. Data storage and handling was compliant with all Kenyan laws and regulations. Confidentiality of research subjects and personnel records was ensured by anonymizing data analysis and encrypting and safeguarding access of all personal information.

## Results

Between September 2016 and April 2017, a total of 2261 persons were screened for potential inclusion in the study (via the outreach program as well as in the three participating clinics) with 289 consenting persons fulfilling the inclusion criteria of fever (> 37.5 °C measured by forehead scanner) or other associated symptoms and access to a Safaricom-operated mobile phone; 1 patient was excluded due to lack of consent. Thus 288 patients were included in the final analysis.

### Patient demographic information

Main patient demographics are described in Table 1. Of patients whose gender was recorded, 59% were female (48% of the entire study population). As is expected within this study population, a large proportion of patients were pastoralists and farmers (39%) and most of them lived in a temporary housing situation (71%), as is characteristic of the nomadic Samburu tribe. The majority of participants had no formal education (54%). Lack of access to transportation to a local clinic was reported by 38% of patients.

**Table 1:** Patient Demographic Information (*N* = 288)

Gender	N(%)
Female	138 (47.9%)
Male	94 (32.6%)
Unrecorded/missing	56 (19.4%)
Age in years	
Mean (SD)	28.1 (17.7)
Median	26.0
Range	[1, 80]
Household position	N(%)
Head	104 (36.1%)
Spouse	77 (26.7%)
Child	100 (34.7%)
Other	7 (2.4%)
Education	N(%)
None	154 (53.5%)
Primary	67 (23.3%)
Secondary	52 (18.1%)
University	15 (5.2%)
Profession	N(%)
Pastoralist	100 (34.7%)
Farmer	13 (4.5%)
Businessman	26 (9.0%)
Civil Servant	16 (5.6%)
Student	66 (22.9%)
Other	6 (2.1%)
None	61 (21.2%)
Housing Status	N(%)
Temporary	203 (70.5%)
Permanent	85 (29.5%)
Self-reported access to transportation to a clinic N(%)	
Yes	178 (61.8%)
No	108 (37.5%)
Unreported/missing	2 (0.7%)

### Patient clinical data

Patient self-reported symptomatology is detailed in Table 2 (for patients under 18 years of age, the parent/guardian was enlisted to report on the child’s symptoms). Fever was a central symptom in this study, with 83% of patients reporting having fever within the prior week. Despite this, only 23% of patients were determined to have fever at time of admission to the study (despite the relatively low threshold of 37.5 °C). Headache was another prevalent symptom reported by patients (86%).

**Table 2:** Patient Symptomatology (*N* = 288)

Symptom	N (%)	Reported duration in days Median (IQR)	Patients reporting recurrence N (%)
Fever (self-reported)	240 (83.3%)	3 (2–7)	219 (91.3%)
Fever (on admission) (> 37.5 °C)	66 (22.9%)	–	–
Night sweats	138 (47.9%)	3 (2–5)	131 (92.9%)
Fatigue	135 (46.9%)	3 (2–5)	124 (91.9%)
Weight loss	104 (36.1%)	–	–
Nausea	117 (40.61)	3 (2–4)	109 (93.2%)
Headache	249 (86.5%)	3 (2–6)	227 (91.2%)
General discomfort	137 (47.6%)	3 (2–5)	120 (87.6%)
Joint pain	137 (47.6%)	3 (2–7)	127 (92.7%)
Lower back pain	125 (43.4%)	3 (2–6)	119 (95.2%)

Of the 288 enrolled patients, 225 consented to testing for brucellosis, and of these 158 consented to testing for malaria. Of those tested for malaria, only 1 patient was diagnosed with malaria (0.6%). Of patients tested for brucellosis, 10 tested positive for Brucella IgG, 9 tested positive for Brucella IgM, and 2 tested positive for both IgG and IgM. Thus, in total 21 patients tested positive for brucellosis antibodies, giving a 9% brucellosis test-positivity rate within this study. All 21 brucellosis-positive patients received vouchers to their mobile phones via the mobile health wallet. These funds were earmarked for transportation to the local clinic (for those who were tested during the outreach program) as well as for brucellosis treatment. For the remaining 204 patients, they received vouchers to their mobile phones earmarked for funding transportation to the local clinic. Of the 21 patients testing positive for brucellosis, demographic, symptom and risk factor information was available for 18 patients, which is detailed in Table 3. Fever and headache were the most prevalent self-reported symptoms of brucellosis patients.

**Table 3:** Patients testing positive for brucellosis (*N* = 21)

Gender	N(%)
Female	11 (61.1%)
Male	7 (38.9%)
Age in years	
Mean (SD)	31.8 (19.6)
Median	26.5
Range	2–68 years
Symptoms	N(%)
Self-reported fever	17 (94.4%)
Fever (on admission) (> 37.5 °C)	10 (55.6%)
Night sweats	10 (55.6%)
Fatigue	5 (27.8%)
Nausea	7 (38.9%)
Weight loss	5 (27.8%)
Headache	16 (88.9%)
Risk Factors	N(%)
Consume raw milk	11 (61.1%)
Consume fresh blood	2 (11.1%)
Consume raw meat	2 (11.1%)
Contact with livestock	16 (88.9%)
Milking	12 (66.7%)
Feeding	12 (66.7%)
Lambing/calving	5 (27.8%)
Slaughtering	6 (33.3%)
Reported livestock health problems within 2 months prior to admission in study (abortion, weak siblings, infected udder, or swelling knee)	11 (61.1%)

### Population risk factors for brucellosis

Within the study population, 88% of patients reported regular contact with livestock. Slaughtering, milking, and lambing/calving of livestock – which increased risk of contracting brucellosis due to the possible contamination from secretions, raw milk and blood – comprised a large proportion of these activities. Self-reported consumption behaviors indicated that 45% of patients consumed raw milk, and 9% consumed fresh blood. Patients consistently reported washing their hands after contact with livestock (90% after milking, 91% after lambing/calving, and 96% after slaughtering livestock). Within the study population 56% of patients indicated that their livestock had suffered from some health problem within 2 months prior to study enrollment, including abortion, infected udder, weak siblings, swellings of the knee, and other problems, which all could be related to brucellosis as well as to other disease causes.

Of patients testing positive for brucellosis, 50% were pastoralists, indicating expected frequent access to livestock. As indicated in Table 3, of the 18 brucellosis-positive patients with risk factor data, 16 reported contact with livestock (milking, feeding, lambing/calving, and slaughtering). Similarly, 61% of brucellosis patients reported that their livestock had suffered from some health problem within 2 months prior to study enrollment. Univariate analyses (Chi-Square tests) indicated that there was no significant association between reported risk factors (being a pastoralist, having contact with livestock, consuming raw animal products, or reporting livestock health problems) and brucellosis diagnosis.

### Patient follow-up

Follow-up phone calls were made to all patients (for patients who were under 18 years of age, follow-up interviews were given to the parent/guardian). Of the total study population, 117 of 204 (57%) of brucellosis-negative patients and 18 of the 21 brucellosis-positive patients were reached. The malaria-positive patient was unable to be located despite repeated efforts. All patients who could be reached for follow-up reported receiving a transport voucher to attend the hospital/clinic. Within the brucellosis-positive group, 88% of patients reported acceptance of mobile heath wallet and that the funds they received in their mobile health wallet covered all treatment costs. Self-reported full treatment adherence within the brucellosis-positive population was 86%, and all patients reached for follow-up reported satisfaction with the program and stated that their health and symptoms improved following treatment. For malaria- and brucellosis-negative patients (who were also given transport vouchers to go to the health facility), only 2 of 117 patients reported still being treated for malaria upon arriving at the health facility.

### Mapping of diagnostic data

**Fig. 2:**
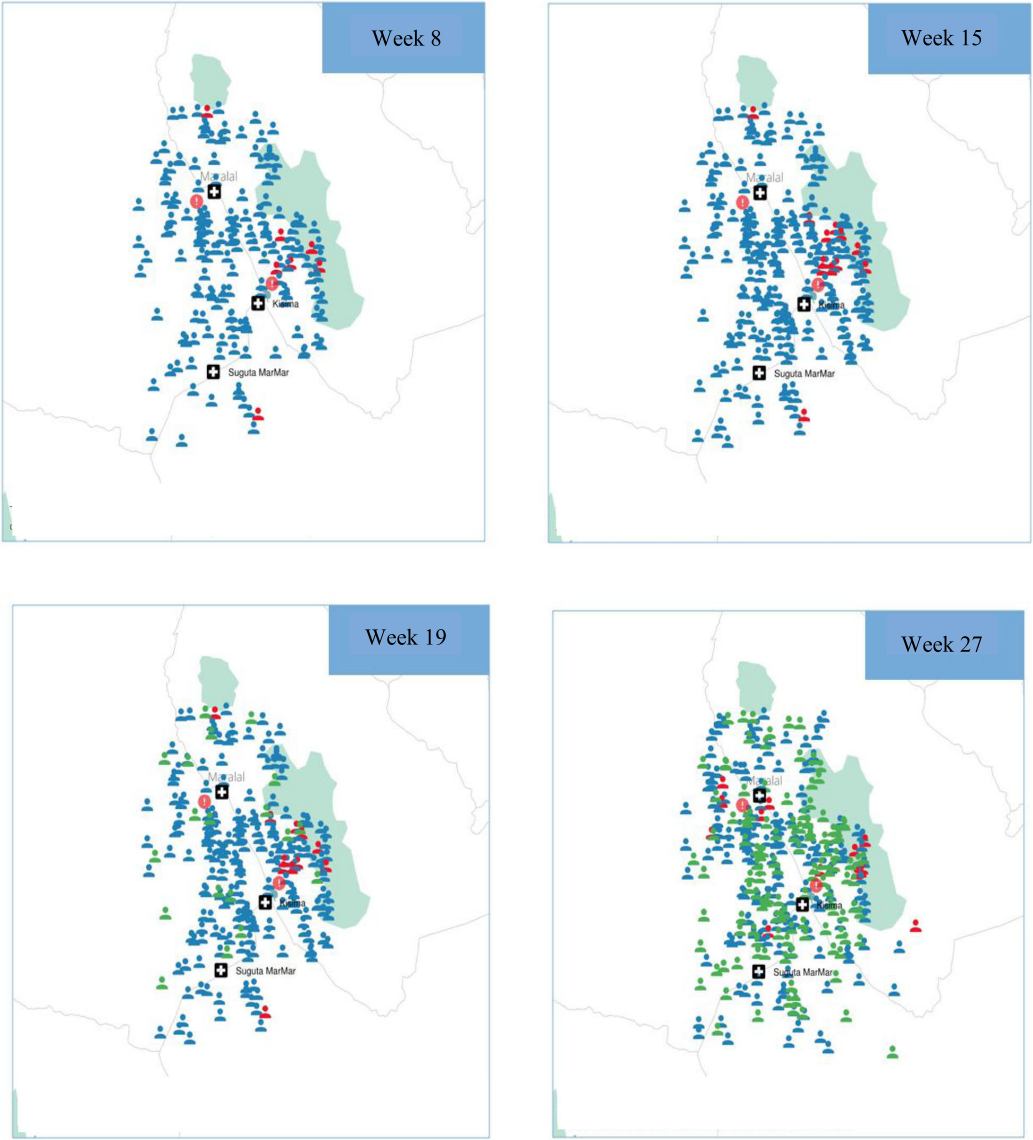
Time lapse of geographic mapping of diagnoses during the course of project implementation. These maps include brucellosis negative (blue human icon) and positive (red human icon) diagnoses across Samburu County, in relation to key risk areas (butcheries, slaughterhouses, etc.) (indicated by exclamation icon) and clinic/hospital locations (indicated by cross icon). Human icons in green demonstrate malaria-negative and brucellosis-negative patients who over time, upon follow-up, reported recovering from their symptoms. All brucellosis patients who could be reached for follow-up reported successful recovery

The use of the Deki reader, which logs longitude and latitude at point of diagnosis, provided the opportunity to generate a map based on GPS coordinates of where each patient was diagnosed. Fig. 2 demonstrates a time-lapsed map of patients across Samburu county who were tested for brucellosis and their respective diagnoses during four randomly-selected weeks of the project.^2^ Positively-diagnosed cases of brucellosis (indicated by red human icons) and negatively-diagnosed cases of brucellosis (indicated by blue human icons) were mapped in relation to the location of high-risk areas for brucellosis transmission, such as slaughterhouses and butcheries (indicated by red exclamation icons). Human icons in green demonstrate malaria-negative and brucellosis-negative patients who over time, upon follow-up, reported recovering from their symptoms. There was no significant association between geographic location of patient diagnoses and key risk locations, as tested by buffering analysis using ArcGIS. This map demonstrates the value of the test reader in determining the location of diagnoses relative to risk factors, which can also be useful for tracking prevalence of brucellosis within the area.

## Discussion

This intervention provided proof-of-principle that a Connected Dx approach, in which POC testing combined with mobile digital diagnostic readers linked to network-connected databases and mobile health wallets, can be successful in the facilitation of brucellosis diagnosis, treatment, and follow-up in a remote area within the Samburu, a nomadic community that presents unique challenges for diagnosis and treatment.

When considering the ongoing issue of malaria overdiagnosis in Africa, this project is a timely example of an approach to improving diagnosis and case management of infectious illnesses in resource-poor contexts. Complications of brucellosis, when left untreated in the long-term, may lead to highly disabling conditions that range from inflammatory arthritis of joints and spine to infections of the central nervous system and other organs. Due to the ambiguous nature of brucellosis symptoms, identifying this illness within a clinical setting in Samburu requires serum testing and the need for equipment and staff that are often limited or unavailable, delaying opportunities for treatment. The ability now offered through Connected Dx to diagnose patients for brucellosis at POC, link this diagnosis directly to treatment via a mobile health wallet, and facilitate telephone follow-up to determine patient outcomes can have substantial positive impact on brucellosis-related morbidity. Furthermore, considering that most diagnosed patients were of working age, we expect that this program positively impacted patients’ capacity to work and earning potential over the long-term, an important outcome that is worth further study in a population that is dependent on physical labour for economic survival. A lesser emphasized outcome is the relatively high level of self-reported treatment adherence seen in response to this intervention. Adherence is particularly important for treatment success and the prevention of drug resistance. Keeping patients connected via their mobile phones through the diagnosis, treatment, and follow-up process undoubtedly contributed to this level of adherence. Furthermore, the incentive of providing funds earmarked for healthcare dispensed to patients via their mobile health wallets likely also contributed to good adherence rates, highlighting the potential for healthcare pre-payment and Universal Health Coverage in promoting positive health-seeking behaviors and treatment adherence.

Connected Dx centered on providing treatment for specific infectious illness based only on correct diagnosis, thereby avoiding misdiagnosis, incorrect treatment, and misuse of drugs. Research has demonstrated that, in some areas of Kenya, as many as 63% of patients presumptively diagnosed and treated with malaria in the clinical setting in fact do not have malaria [48]. Upon examining the Samburu County Health Facility data for the year prior to implementation of this study (including pharmacy records, clinical records, and lab records), we determined that clinical diagnosis of malaria without laboratory confirmation occurred in 42% of reported malaria cases in the three health facilities included in this study (that is, for 42% of antimalarial drug regimens dispensed in the clinic pharmacy, there was no corresponding positive malaria test) [27]. The Connected Dx approach implemented in this study resulted in the detection of only 1 malaria case out of 158 tested patients (0.6%), and only 2 of 117 patients reported that they had been treated for malaria upon arriving at the health facility (despite both testing negative for malaria via RDT)). These results, compared to the County Health Facility data, highlights the potential of Connected Dx (an infectious disease case management approach requiring the dispensing of drugs to be directly linked to a positive test) for the reduction of malaria overtreatment in clinical settings.

This study did face some challenges and limitations. Drug availability in some dispensaries and clinics was at times limited. Due to the small number of patients testing positive for brucellosis, we were unable to conduct robust analyses on the prevalence of brucellosis and its linkage to key risk factors within the Samburu population. Despite Samburu being a rural county, its estimated mobile penetration of 77% indicated that a mHealth approach could be readily implemented. However, several challenges were encountered throughout implementation. Mobile phones were often shared within households and some communities had limited mobile network availability, limiting follow-up efforts (40% of patients could not be reached for follow-up). This loss-to-follow-up, while likely unavoidable in a nomadic population such as the Samburu, prevents us from determining the true impact of Connected Dx on treatment and adherence. Furthermore, the lower literacy level of the population rendered difficulty in the usage of the mHealth wallet for some patients.

The fact that the Connected Dx intervention could work even when addressing a remote, nomadic population indicates that this approach to infectious disease diagnosis, treatment, and case management can be scaled to more prevalent diseases and conditions in more populous contexts. The Deki reader can be calibrated to support additional rapid diagnostic tests commercially available, including HIV and malaria [41]. Currently M-TIBA, a Kenyan-operated mobile health wallet that has already connected approximately 4 million users and nearly 900 healthcare providers in Nairobi and Kisumu Counties, is widely expanding, providing a more suitable, health specific wallet than which was used in this project in the context of Samburu [49]. Within this project funding of travel and treatment vouchers via the mobile health wallet was provided by a grant from Achmea Foundation; however, we believe Kenya’s recent commitment to universal healthcare coverage [48] indicates the potential for local funding of Connected Dx approaches to diagnosis and treatment of infectious diseases that can be detected via RDT. The success of this project presents the opportunity to establish a new norm for accurate diagnosis and treatment of febrile diseases in both remote and urban populations. Today, a similar Connected Dx approach is being implemented for the diagnosis and treatment of malaria in clinics in Kisumu County, Kenya, which has one of the highest malaria prevalence rates in Kenya and an estimated high malaria misdiagnosis rate [50].

## Conclusion

This study, through demonstrating that a Connected Dx approach can be used to provide accurate diagnosis and guide conditional payments for treatment of brucellosis, has provided a strong basis for continued future iterations to address additional infectious illness that can be digitally diagnosed at POC. The shift from presumptive or inadequate diagnostic procedures to one that is digitalized, accurate, streamlined for quality assurance and supports targeting of funds to those patients who are in need will contribute to improved health outcomes and cost savings overall.

## Data Availability

Due to privacy of patient data, datasets associated with this project are not publicly available. Anonymized data is available from the authors upon reasonable request.

## Abbreviations

CHV: Community Health Volunteer
CRF: Case record form
HIV: Human immunodeficiency virus
NHIF: National Hospital Insurance Fund
POC: Point-of-care
RDT: Rapid diagnostic test
SMS: Short messaging services
TB: Tuberculosis
WHO: World Health Organization

## Footnotes

1
https://www.dimagi.com/commcare/

2 An interactive version of this map can be viewed at: https://www.google.com/maps/d/viewer?mid=164Fmp7ajQavjJ__vYbroZjfUASA&ll=0.9082967175105664%2C36.776445388793945&z=11
